# Computerized Adaptive Testing for Schizotypal Personality Disorder: Detecting Individuals at Risk

**DOI:** 10.3389/fpsyg.2020.574760

**Published:** 2021-01-25

**Authors:** Yaling Li, Menghua She, Dongbo Tu, Yan Cai

**Affiliations:** School of Psychology, Jiangxi Normal University, Nanchang, China

**Keywords:** computerized adaptive testing, schizotypal personality disorder, schizotypy, item response theory, assessment

## Abstract

As schizotypal personality disorder (SPD) increasingly prevails in the general population, a rapid and comprehensive measurement instrument is imperative to screen individuals at risk for SPD. To address this issue, we aimed to develop a computerized adaptive testing for SPD (CAT-SPD) using a non-clinical Chinese sample (*N* = 999), consisting of a calibration sample (*N*_1_ = 497) and a validation sample (*N*_2_ = 502). The item pool of SPD was constructed from several widely used SPD scales and statistical analyses based on the item response theory (IRT) *via* a calibration sample using a graded response model (GRM). Finally, 90 items, which measured at least one symptom of diagnostic criteria of SPD in the fifth edition of the Diagnostic and Statistical Manual of Mental Disorders (DSM-5) and had local independence, good item fit, high slope, and no differential item functioning (DIF), composed the final item pool for the CAT-SPD. In addition, a simulated CAT was conducted in an independent validation sample to assess the performance of the CAT-SPD. Results showed that the CAT-SPD not only had acceptable reliability, validity, and predictive utility but also had shorter but efficient assessment of SPD which can save significant time and reduce the test burden of individuals with less information loss.

## Introduction

Schizotypal personality disorder (SPD) is a serious psychiatric disorder, characterized by a pervasive pattern of social and interpersonal deficits marked by acute discomfort with, and reduced capacity for, close relationships (Lentz et al., 2010). It is also related to cognitive or perceptual distortions and eccentricities of behavior, beginning with early adulthood and presenting in a variety of contexts in the fifth edition of the Diagnostic and Statistical Manual of Mental Disorders (DSM-5; American Psychiatric Association [APA], 2013). Although rarely seen in clinical practice, an epidemiologic study using an American sample found that the lifetime prevalence of SPD is 4.2% among men and 3.7% among women (Pulay et al., 2009). In addition, SPD is widely recognized as an early-onset state of schizophrenia spectrum disorder (Zhang, 2014). Long-term follow-up studies found that 25% patients with SPD have a great potential to develop schizophrenia (Asarnow, 2005). Most studies in the area of psychosis suggested that early identification, prevention, and intervention can bring benefits for adolescents who have a higher probability to develop psychosis from the general population, such as mitigating the impact of disease on personality, work, and social interaction (Yung et al., 2007; McGorry et al., 2008). To facilitate early detection and intervention, a reliable and valid measurement instrument is essential for identify young people at-risk for psychosis.

Psychometric detection of individuals with high risk of developing schizophrenia spectrum disorders is a critical enterprise. Recently, many self-report measurements for SPD have been developed for this purpose, such as the Schizotypal Personality Questionnaire (SPQ; Raine, 1991), the Structured Interview for Schizotypy (SIS; Kendler et al., 1989), the Oxford-Liverpool Inventory of Feelings and Experiences (O-LIFE; Mason et al., 1995), etc. However, it is difficult for a single scale to reveal the whole picture of SPD. For example, the O-LIFE only measures four of the nine symptoms (Furnham et al., 2014). The SPQ (Raine, 1991), which mirrors nine schizotypal traits of SPD that are laid out in the DSM-4, has a widespread application in clinic practice. There are some shortcomings with this instrument. First, it has 74 items, which would increase test burden and decrease test motivation. Second, based on the assumption that 10% of the population suffers from schizotypy (Meehl, 1962; Lenzenweger and Korfine, 1992; Lenzenweger, 2006), the top 10% of scores on the SPQ sum score are classified as having SPD. The way of classification that categorizes the top 10% scores on the SPQ having SPD is inappropriate for some specific population, which is ascribed to the fact that there are enormous discrepancies among different populations.

Despite this, it should be noted that they were developed under the classical test theory (CTT) frame and had some drawbacks. For example, in order to ensure comparability of scores, all participants were asked to administer the same items, which meant the questionnaire might not be invariably optimal: some participants have to administer items which are not suitable for their latent trait, and some domains or factors of test may be measured with less accuracy than is desired. The item response theory (IRT) was developed as an alternative to CTT to overcome these shortcomings. With the advancement of computer technology and the rapid development of IRT, computerized adaptive testing (CAT) as a new measurement technique was developed under the framework of IRT over recent decades. It is considered to be a suitable measurement method for various types of psychological assessments in that CAT has several merits than traditional measurement instruments. First, CAT chooses items based on test-takers’ immediate ability estimators, which can skip questions not necessary for them. Second, CAT replaces traditional reliability with the test information function of different trait levels, which implies that we can actively operate each individual’s measurement error by controlling the test information.

Schizotypal personality disorder as a fairly stable and highly disabling disorder (Pulay et al., 2009) not only negatively affects individuals and even their families but also is related to significant mental disorder comorbidity and low quality of life (Lentz et al., 2010). In a clinical setting, accurate and immediate detection and diagnosis is crucial for the treatment of a disease, and SPD as one of the psychosis spectrum disorders is no exception. When the measurement tool is administered with an adaptive version, the rise in measurement precision on certain psychological traits (Jacobusse and van Buuren, 2007) and the efficiency on the detection of a certain disease/disorder took place (Smits et al., 2011). Thus, this paper aims to incorporate a computer-based adaptive test, the CAT, to advance the field of SPD assessment. With regard to CAT for schizotypy, there are different versions. For example, Moore et al. (2018) developed a fixed-length CAT version for SPQ. Fonseca-Pedrero et al. (2013) developed a CAT based on the Oviedo Schizotypy Assessment Questionnaire (ESQUIZO-Q; Fonseca-Pedrero et al., 2010), which is a self-rating scale consisting of 51 items in a five-point Likert-type response format.

In spite of different CAT versions that have already been developed, there are still some issues that need to be further settled. First of all, existing CAT versions (e.g., Fonseca-Pedrero et al., 2013; Moore et al., 2018) were constructed based on only one questionnaire, which is difficult to reveal the whole picture of SPD. Second, methodologically, there are many IRT models that may fit different data types under the IRT frame. Nevertheless, few studies have chosen an optimal model to fit the CAT based on its data. Third, there are some obvious weaknesses in previous researches. For instance, in the thesis of Moore et al. (2018), the fixed-length CAT was used, which typically leads to different measurement precisions between test-takers with diverse trait levels. Larger measurement errors will occur in individuals with extreme trait levels (Choi et al., 2010). In the study of Fonseca-Pedrero et al. (2013), the graded response model (GRM; Samejima, 1969), a widely used polytomous IRT model, was used to calibrate items when the item pool was multidimensional, which may negatively affect the psychometric functioning of the CAT based on the unidimensional assumption. More importantly, a research study investigating 21 cities in China showed that the positive check rate of SPD was 15.5% for male undergraduates and 9.3% among female undergraduates (Ling et al., 2008). Hence, it is imperative to construct an effective CAT covering all aspects of SPD in China. The objective of this study is to develop a new, more efficient CAT for SPD which overcomes the abovementioned drawbacks. The research will thus be capable of considerately advancing the field of SPD assessment.

## Materials and Methods

### Participants

A total of 1,127 university students were enrolled from seven cities of China in this research. All participants engaged voluntarily without any payment. The questionnaire comprised some basic demographic questions, items for SPD, and excluding criteria. To strike out individuals with random responses, three lie detection items which were developed to opposite meanings according to three SPD items were placed in the questionnaire. An original item for SPD includes the question “Do you feel nervous when someone follows you”? The corresponding lie detection item was “I am not nervous when someone follows me.” Subjects having the same responses to any of the three pair items were removed for this research.

Of those, 5.7% (*N* = 64) respondents were excluded because of lie detection items; 1.3% participants (*N* = 15) were eliminated due to satisfying any of the preset excluding criteria presented as follows: (1) prior diagnosis of psychiatric disorders; (2) prior diagnosis of brain organic disease caused by infection, tumor, and trauma; (3) prior diagnosis of cognitive impairment or mental deficiency; and (4) experiencing events having a great impact in the past 1 month (Yunfei et al., 2006).

Besides, 4.3% (*N* = 49) were partial completers, and most missing value appeared in the demographic variables. Hence, the MissMech R package was applied to test whether the data are missing completely at random (Rubin, 1976). Based on the result of the test supporting that the data is missing completely at random and the lower percentage of missing value (4.3 < 10%; Bennett, 2001), missing data were removed using the method of list-wise deletion. Therefore, the final valid sample contains 999 subjects (88.6%). The current study was carried out following the recommendations of psychometrics studies on mental health at the Research Center of Mental Health of Jiangxi Normal University. Informed consent was obtained from all participants in accordance with the Declaration of Helsinki.

Applying the same sample to both calibrate items and to simulate CAT may lead to capitalization on chance providing optimistic outcomes (Smits et al., 2011), and the cross-validation sample can be used to deal with this problem (Stone, 1974). Therefore, we decided to divide the sample of 999 participants into two random and independent subsamples: the calibration sample (*N* = 497) and the validation sample (*N* = 502). The calibration sample was employed to construct the item pool for the CAT-SPD and calibrate item parameters of the final item pool, while the validation sample served to explore the psychometric characteristics of the CAT-SPD.

Table 1 shows detailed demographic information of both samples. For the calibration sample, 63.98% are female. Besides, 54.33% of the sample comes from the rural area and 26.16% are only one child. As for grade, the distribution was as follows: 60.8% freshman and sophomore, 30.5% junior and senior, and 8.8% postgraduate. The mean age was 20.56 (*SD* = 1.85, range 16–29), and 95.8% of the participants were between 16 and 24 years of age. With regard to the validation sample, a similar pattern was observed on the demographic variables concerning gender, region, one child, grade, and age.

**TABLE 1:** Demographic characteristics of the calibration sample and the validation sample.

Characteristic	Validation sample, % (*N*_1_ = 502) Total (male/female)	Calibration sample, % (*N*_2_ = 497) Total (male/female)
**Gender** Male Female	39.04 60.96	36.02 63.98
**Region** Unban Rural	46.61 (37.77/62.23) 53.38 (40.15/59.85)	45.67 (35.24/64.76) 54.33 (36.67/63.33)
**One child** Yes No	25.89 (42.31/57.69) 74.10 (37.90/62.10)	26.16 (46.92/53.08) 73.84 (32.15/67.85)
**Grade** Freshman and sophomore Junior and senior Postgraduate	58.80 (42.30/57.70) 32.60 (39.22/60.78) 8.70 (15.91/84.09)	60.80 (34.93/65.09) 30.50 (41.36/58.64) 8.80 (23.36/76.74)
**Age** 16–20 21–24 25–29	58.40 (40.27/59.73) 36.90 (38.92/61.08) 4.80 (25.00/75.00)	59.60 (33.78/66.22) 36.20 (40.56/59.44) 4.20 (28.57/71.43)

### Measurement

In this study, three well-validated scales of SPD were used to be the source of the original item pool, including the SPQ, the Referential Thinking Scale (REF; Lenzenweger et al., 1997), and the Five-Factor Schizotypal Inventory (FFSI; Edmundson et al., 2011). The SPQ is a self-evaluation scale with a binary answer of “yes” or “no.” It has been proven that the SPQ has adequate reliability and validity in many articles (e.g., Raine, 1991; Furnham et al., 2014). The REF that measures the sample and guilty ideas of reference is a unidimensional questionnaire, composed of 34 items. As for reliability, the original paper cites that Cronbach’s alpha and test–retest reliability are 0.83 and 0.86 (Furnham et al., 2014). With respect to validity, high REF scores were associated with increased levels of schizophrenia-related psychological deviance (Lenzenweger et al., 1997). The FFSI, which measures schizotypy from the prospective of the five-factor model of general personality structure, includes nine subscales, with 10 items per subscale. The FFSI showed good psychometric properties, embodied in the subscales’ coefficients of Cronbach’s alpha ranging from 0.82 to 0.94, and the convergence validity is up to 0.77 (Edmundson et al., 2011). All three scales served to measure SPD, with each measure offering various levels of or focusing on specific dimensions (Furnham et al., 2014).

We carefully selected items that at least measure one diagnostic criterion of SPD in the DSM-5 from those scales to form the initial item pool. Finally, 128 items that met the above criterion were selected to make up the original item pool. Seventy-four items came from the SPQ. The remaining 54 items were from the other two scales. In order to ensure each symptom can be comprehensively measured, we tried to select at least 10 items per diagnostic criterion.

The Personality Diagnostic Questionnaire-4 (PDQ-4; Hyler, 1994), designed to assess all 10 of the DSM-IV personality disorders, served as a criterion scale to evaluate the validity of the CAT-SPD.

### Statistical Analysis

Statistical analysis comprised two sections: development of an item pool for CAT-SPD and the simulation study of the CAT-SPD. IRT analyses of the former section were conducted with the calibration sample. The later section was carried out with the validation sample.

#### Development of the Item Pool for CAT-SPD

Step 1: Test unidimensionality of the initial item pool

Although a unidimensional item pool is not a precondition for CAT, most IRT models consider unidimensionality as a fundamental assumption. It implies that responses to each item are affected by a single latent construct of test-takers. Exploratory factor analysis (EFA) and confirmatory factor analysis (CFA) were used simultaneously to evaluate the unidimensionality of the item pool. In EFA, the rations of total variance explained by the first factor are above 20% (Reckase, 1979) and the value of the first eigenvalue divided by the second eigenvalue is equal to 4 or higher, which is commonly accepted to support the assumption of unidimensionality (Reeve et al., 2007). In CFA, given that some items were binary indicators, we used weighted least square means and a variance (WLSMV)-adjusted estimation, which has a more accurate estimation when the variables are categorical data (Beauducel and Herzberg, 2006; Resnik et al., 2012). If the comparative fit index (CFI) ≥ 0.85, the Tucker–Lewis index (TLI) ≥ 0.85, and the root-mean-square error of approximation (RMSEA) ≤ 0.08, the model is judged as acceptable (Hu and Bentler, 1999). First, we conducted EFA based on 128 items and removed items with the first loading less than 0.3 to ensure sufficient unidimensionality of the item pool. Then, EFA and CFA were conducted to evaluate the unidimensionality of the remaining items in the item pool. This process was conducted till the remaining items were unidimensional.

Step 2: Select the appropriate IRT model

The fit of the parametric IRT model is extraordinarily momentous in the implementation of IRT (Liang and Wells, 2009). In the current study, four widely used polytomous models were considered: GRM, generalized partial credit model (GPCM; Muraki, 1997), partial credit model (PCM; Masters, 1982), and nominal response model (NRM; Bock, 1972). Then, the most suitable model was chosen *via* three test-level model-fit indices: –2log-likelihood (-2LL; Spiegelhalter et al., 1998), Akaike’s information criterion (AIC; Akaike, 1974), and Bayesian information criterion (BIC; Schwarz, 1978). The smaller the values of those indices, the better the fit of the model. Thus, the model with the smallest -2LL/AIC/BIC was selected and employed for subsequent analyses.

Step 3: Evaluate the local independence of the remaining items

Local independence is the underlying assumption of the IRT models. It implies that, given an individual’s score on the latent variable, responses to each item should be independent with other items in the same test (Embretson and Reise, 2000). A *Q*_3_ statistic proposed by Yen (1993) was used to detect local independence and the *Q*_3_ value above 0.36 represents dependence (Flens et al., 2017). The item pairs with coefficient over 0.36 were labeled. Then, an item with a larger cumulative *Q*_3_ was removed from the item pool.

Step 4: Check the item model fit of the remaining items

Testing of goodness of item fit is proven to be an important step when conducting IRT-based analysis (Köhler and Hartig, 2017). The *S-X*^2^ statistic was used to test the item fit. According to Flens et al. (2017), items with a *p*-value of *S-X*^2^ less than 0.001 were considered as a misfit. A sterner criterion was applied. Items whose *p-*value of *S-X*^2^ was less than 0.01 were deleted.

Step 5: Choose items with high discrimination parameter

The discrimination parameter in IRT is a critical index to assess the quality of items. Chang and Ying (1996) suggested that a value between 0.5 and 2.5 for discrimination was deemed acceptable. In this study, items with discrimination below 0.5 were deleted from the item pool to form a high-quality item pool for SPD.

Step 6: Assess differential item functioning (DIF) of the remaining items

To build a non-biased item pool, DIF (Embretson and Reise, 2000) analysis was conducted to identify whether an item has a measurement bias due to demographic variables such as gender (female, male) and region (urban, rural) (Gaynes et al., 2002). The ordinal logistic regression (Crane et al., 2006) method was applied to perform DIF analysis. The change of McFadden’s pseudo-*R*^2^ was employed to assess effect size. The change of *R*^2^ is greater than 0.02 (Flens et al., 2017), indicating that the item is biased and should be considered for deletion. This criterion was applied to decide whether an item should be removed.

##### Summary

The study conducted successively IRT analyses including unidimensionality, model selection, local independence, item model fit, discrimination, and DIF. Only if an item met all measurement requirements could it be retained in the final item pool: (1) measuring at least one diagnosis criterion of SPD in the DSM-5, (2) meeting the unidimensionality assumption, (3) keeping local independence, (4) fitting the IRT model, (5) possessing high discrimination higher than 0.5, and (6) having no DIF. After completing the above steps, item parameters were re-estimated for the subsequent analyses.

#### A Simulated CAT for CAT-SPD

A simulated CAT was conducted in the validation sample based on real data, which aimed to investigate the performance of the CAT-SPD, including the characteristics, marginal reliability, criterion-related validity, and predictive utility (sensitivity and specificity) of the CAT-SPD.

##### Starting level

The item selection in CAT relies on participants’ response to previous items. Yet, the respondent knows nothing about prior information in the initial stage of a test (Kreitzberg and Jones, 1980). Choosing an item randomly from the final item pool is an effective and uncomplicated method (Magis and Barrada, 2017). The study applied this method to start the CAT.

##### Estimating score

In the development of IRT, many psychometricians had proposed many estimation methods of latent trait: (1) maximum likelihood estimation (MLE) method (Rasch, 1993), (2) weighted maximum likelihood (WLE) method (Warm, 1989), and (3) expected *a posteriori* (EAP) method (Bock and Mislevy, 1982). Here, the EAP was employed to estimate test-takers’ latent trait given that it utilizes prior information of latent variable, needs no iteration, and estimates latent variable with high accuracy (Bock and Mislevy, 1982). The formula of EAP is defined as,

$${\hat{\theta }}_{i}=\frac{\sum_{\text{h}=1}^{q}Z_{h}\,L_{i}\,\left(Z_{h}\right)\,W\,\left(Z_{h}\right)}{\sum_{h=1}^{q}L_{i}\,\left(Z_{h}\right)\,W\,\left(Z_{h}\right)},$$

where *Z*_*h*_ means the quadrature points as an alternative value for the specific theta. *L*_*i*_(*Z*_*h*_) is the likelihood function of participant *i* with a specific response pattern. *W*(*Z*_*h*_) is the weight of the quadrature point of *Z*_*h*_.

##### Item selection strategy

The CAT algorithm chooses the next item providing maximum information given interim estimator, which is known as the maximum Fisher information (MFI) criterion (Baker, 1992). MFI is related to the measurement error of the estimated latent variable. The greater the amount of information provided by an item, the higher the accuracy of the trait estimated. The Fisher information is expressed as follows:

$$I_{j}\,\left(\hat{\theta }\right)=\sum_{k=1}^{K}\frac{{\left[P_{k}^{\prime }\,\left(\hat{\theta }\right)\right]}^{2}}{P_{k}\,\left(\hat{\theta }\right)},$$

where $I_{j}\,\left(\hat{\theta }\right)$ is the item information function of item *j* at current estimated $\hat{\theta }$. $P_{k}\,\left(\hat{\theta }\right)$ is the probability of the receiving score *k* given $\hat{\theta }$. *K* is the sum score of item *j*, and $P_{k}^{\prime }\,\left(\hat{\theta }\right)$ is the first derivative of $P_{k}\,\left(\hat{\theta }\right)$ to $\hat{\theta }$. The MFI method was used to select an item for the CAT-SPD to improve the accuracy of measurement.

##### Stopping rule

Computerized adaptive testing termination strategies can be divided into two main categories: fixed length and variable length. The former means terminating the test when the number of items administered has reached a fixed value. The latter refers to ending the test when the predefined level of measurement precision has been met. A fixed-length stopping rule might limit the effectiveness of adaptive tests through assigning unsuitable items that contribute little to the subject’s level of trait (Choi et al., 2010). Two types of variable-length termination strategies have been used in a previous study (Dodd et al., 1993), namely, the standard error stopping rule (*SE*) and the minimum information terminating rule (*MI*). In this article, the *SE* was used which is inversely proportional to the test information function.

$$S\,E\,\left({\hat{\theta }}_{i}\right)=\frac{1}{\sqrt{\sum_{j=1}^{n}I_{j}\,\left(\hat{\theta }\right)}}$$

where *n* is the number of administered items for a specific respondent. According to the formula of reliability under the IRT framework: *reliability* = 1 - *SE*^2^ (Fliege et al., 2005). Three stopping rules were set at *SE* ≤ 0.447, 0.386, and 0.316, respectively, which correspond to reliabilities of *r* ≥ 0.8, 0.85, and 0.9, respectively. Simultaneously, the maximum number of selected items was set at 50 to increase the efficiency for each individual (Flens et al., 2017).

##### Characteristics of the CAT-SPD

Several statistics were computed respectively for different stopping rules to investigate the characteristics of the CAT-SPD: the mean and standard deviation (*SD*) of items administered, the mean *SE* of trait estimator, the Pearson’s correlation of estimated trait between each terminating criterion and the full item pool, and the marginal reliability which is the average of all individuals’ reliability (Smits et al., 2011). In the IRT frame, the reliability for each individual can be obtained by the formula (Samejima, 1994),

$$r\,\left(\theta_{i}\right)=1-\frac{1}{I\,\left(\theta_{i}\right)},$$

where *r*(θ*_*i*_*) is the corresponding reliability for the *i*th examinee. Finally, the number of answered items with the test information for the final theta estimation under each stopping rule was plotted to examine the efficiency of the CAT-SPD.

##### Criterion-related validity and predictive utility of the CAT-SPD

When the CAT-SPD estimation result has great consistencies with the result of the well-validated scales, the CAT-SPD might work. In other words, compared with others diagnosed with no SPD, a participant diagnosed with SPD in a scale will have a larger trait estimator in CAT. The consistencies were assessed by criterion-related validity and predictive validity of the CAT-SPD. Criterion-related validity of the CAT-SPD was accessed by Pearson’s correlation between the estimated trait *via* the CAT-SPD and score of schizotypal subscales in PDQ-4.

The receiver operating characteristic (ROC), which takes sensitivity as the ordinate and 1—specificity as the abscissa, is usually applied to evaluate the diagnosis effect in CAT (Lusted, 1960; Smits et al., 2011). In this study, sensitivity means the possibility that a respondent with SPD is correctly diagnosed with SPD, while specificity refers to the possibility that a normal examinee is correctly diagnosed with no SPD. The higher the quantity of sensitivity and specificity, the better the effect of the diagnosis. The area under the curve (AUC) refers to the area under the ROC curve. The larger the AUC, the higher the diagnostic accuracy. In other words, the closer the value of AUC to 1, the better the diagnosis effect. Hence, predictive utility was examined by the AUC, sensitivity, and specificity (Smits et al., 2011).

### Software

The EFA was carried out by SPSS 23.0 and the CFA was conducted by using Mplus 7.0 (Muthén and Muthén, 2012). Other analyses of the CAT-SPD item pool development were performed in R package of *mirt* (Versions 1.24; Chalmers, 2012) and *lordif* (Versions 0.3-3; Choi, 2015) and a simulated CAT was implemented by R self-programming (version 3.4.1; Core Team, 2015).

## Results

### Development of the Item Pool for the CAT-SPD

#### Unidimensionality

The initial item pool with 128 items was run *via* EFA, and 23 items were removed due to their first load less than 0.3. After eliminating 23 items from the item pool, EFA and CFA were conducted based on the remaining 105 items. The result of the one-factor model EFA showed the ratio of the first eigenvalue to the second eigenvalue was equal to 4.47 and many items loaded highly on the first factor that accounted for 20.2% of the total variance. The result of one-factor model CFA showed acceptable model fit: CFI = 0.869, TLI = 0.867, and RMSEA = 0.038. These results indicated that the remaining items of the item pool basically satisfy the unidimensionality assumption.

#### Model Selection

Table 2 presents the model data fit indices, including AIC, BIC, and -2LL, for the four IRT models. Three fitting statistics of the GRM model were the smallest among the four models, implying that the GRM model fitted the data best compared with others. Thus, GRM was selected for the subsequent analyses.

**TABLE 2:** Test-level model fit for the four polytomously scored IRT models.

Model	-2LL	AIC	BIC
GRM	76,659.82	77,283.83	78,596.91
GPCM	77,962.96	78,337.96	79,254.35
PCM	76,943.78	77,567.78	78,880.86
NRM	76,672.40	77,500.40	79,242.76

*-2LL, -2log-likelihood; AIC, Akaike’s information criterion; BIC, Bayesian information criterion; GRM, graded response model; GPCM, generalized partial credit model; PCM, partial credit model; NRM, nominal response model.*

#### Local Independence

A total 11 pairs of items show local dependence in that their absolute *Q*_3_ values were above 0.36. Hence, 11 items with higher cumulative *Q*_3_ were removed from the current item pool.

#### Item Discrimination Parameters

All 94-item discrimination parameters were larger than 0.5. Item 102 owns the largest discrimination parameter (*a* = 2.37), while the discrimination value of item 18 was the lowest (*a* = 0.57). None of the items was removed from the current item pool at this step.

#### Item Model Fit

In the calibration of the remaining 94 items, all items’ *p*-values of *S-X*^2^ were larger 0.01, which indicated that all the remaining items fitted the GRM well.

#### Differential Item Functioning

Of the two group variables (gender, region), there were only four DIF items for the gender variable whose values of *R*^2^ change were 0.025, 0.03, 0.048, and 0.046, respectively. Thus, four items were removed from the current item pool.

#### Summary

In conclusion, we deleted 23 items from the initial item pool to meet the assumption of unidimensionality. Then, in the checking of local independence, 11 items were removed. Finally, four items having significant DIF were screened out from the remaining item pool. After removing these items, the remaining items were reanalyzed for the above processes. We found that none of the items needs to be removed. Therefore, the final item pool for the CAT-SPD consisted of 90 items. Some IRT statistics, providing information from which scale they were based on and the abbreviated content for each item of the final item pool, are partly presented in Table 3 and those of the whole item pool are provided in the Supplementary Material. For the item pool of CAT-SPD, the average discrimination was 1.22 (*SD* = 0.41), which implied the final item pool had high quality. The location parameter ranged from –3.26 to 5.19, which indicates the location parameter had a wide range and basically covered the most values of the traits.

**TABLE 3:** IRT statistics of part item in the final item pool of the CAT-SPD.

Item	Abbreviated item content	Scale	Item parameter					Item-fit estimates			*R*^2^ change	Diagnostic criterion
			Slope	b1	b2	b3	b4	*S-X*^2^	*df*	*p*	DIF	
2	Avoid crowds due to anxiety	SPQ	0.58	–0.07	–	–	–	72.17	80	0.722	0.0052	Social anxiety
4	Mistaken objects for people	SPQ	0.61	1.29	–	–	–	56.87	73	0.918	0.0001	Unusual per experience
5	See me as eccentric	SPQ	1.69	1.40	–	–	–	32.84	43	0.869	0.0025	Odd behavior
7	Hard understand my word	SPQ	1.45	0.63	–	–	–	60.27	64	0.609	0.0024	Odd speech
8	Someone feels I am cold	SPQ	0.82	0.61	–	–	–	73.61	74	0.491	0.0021	Constricted affect
9	Sure I being talked behind me	SPQ	1.07	1.42	–	–	–	48.54	59	0.833	0.0008	Suspicious
10	Fell like people notice me	SPQ	0.87	1.33	–	–	–	51.59	67	0.918	0.0015	Ideas reference
11	Get nervous interacting with others	SPQ	0.70	0.70	–	–	–	72.97	74	0.512	0.0006	Social anxiety
13	Sense force around you	SPQ	0.89	1.40	–	–	–	71.50	65	0.271	0.0036	Unusual per experience
14	Comment my unusual mannerisms	SPQ	1.16	0.72	–	–	–	73.14	67	0.283	0.0124	Odd behavior
15	Keep myself to myself	SPQ	1.53	1.47	–	–	–	37.63	43	0.703	0.007	No friends
87	Feel my body unusual	FFSI	1.17	–1.04	0.29	1.74	3.76	130.11	126	0.383	0.0014	Unusual per experience
88	Think my action odd	FFSI	1.42	–0.04	1.39	2.67	3.20	105.22	89	0.115	0.0106	Odd behavior
91	Don’t form strong bonds	FFSI	1.38	–0.65	0.82	1.59	2.99	112.38	116	0.578	0.0001	No friends
92	Have little to do with other	FFSI	1.21	–0.43	0.89	2.01	3.63	106.96	120	0.797	0.0018	Constricted affect
93	Feel body becoming misshapen	FFSI	1.66	–0.20	0.80	1.66	2.79	104.48	109	0.605	0.0015	Unusual per experience
94	Have odd thinking	FFSI	1.48	–1.08	0.03	1.15	2.54	162.96	135	0.051	0.001	Magic thinking
97	I like to be alone	FFSI	1.10	–2.06	–0.79	0.74	2.72	159.71	134	0.064	0.0131	No friends
98	Describe my behaviors as unusual	FFSI	2.19	-0.43	0.79	1.63	2.87	78.69	90	0.797	0.0025	Odd behavior
100	Feel uneasy with familiar people	FFSI	1.90	-0.59	0.92	1.56	2.53	104.70	100	0.354	0.0093	Social anxiety
101	Sense sometimes is odd	FFSI	1.88	-0.51	0.83	1.61	2.87	119.00	101	0.107	0.0012	Unusual per experience

*SPQ, Schizotypal Personality Questionnaire; FFSI, Five-Factor Schizotypal Inventory.*

### A Simulated CAT for the CAT-SPD

#### Characteristics of CAT-SPD

Table 4 displays the results of the CAT-SPD with individuals’ real response under different stopping rules. The mean number of items administered to individuals is 16.56 (*SD* = 9.50) under the stopping rule reliability ≥ 0.90. If the terminating rule was set up to reliability ≥ 0.85, the average number of selected items is approximately 10.39 (*SD* = 7.57) and then declines further to 7.83 (*SD* = 6.29) when reliability ≥ 0.80. The Pearson’s correlation between the trait estimators in the different terminating rules of CPA-SPD and the trait estimators by the whole item pool ranged from 0.85 to 0.95, which implies that, though a considerable saving item, an accurate estimation of the latent trait is still possible.

**TABLE 4:** Characteristic of the CAT-SPD under several stopping rules.

Stopping rule	Number of items used		Mean *SE* (θ)	Marginal reliability	Cor
	Mean	*SD*			
None	90	0	0.19	0.96	1.00***
Reliability ≥ 0.80	7.83	6.29	0.41	0.83	0.85***
Reliability ≥ 0.85	10.39	7.57	0.36	0.87	0.87***
Reliability ≥ 0.90	16.56	9.50	0.30	0.92	0.95***

***shows the discrepancy on 0.001 levels being notable. None, the whole item bank was administered. Cor refers to Pearson’s correlation of the trait estimations of participants between the whole item pool and different terminating rules.

**FIGURE 1:**
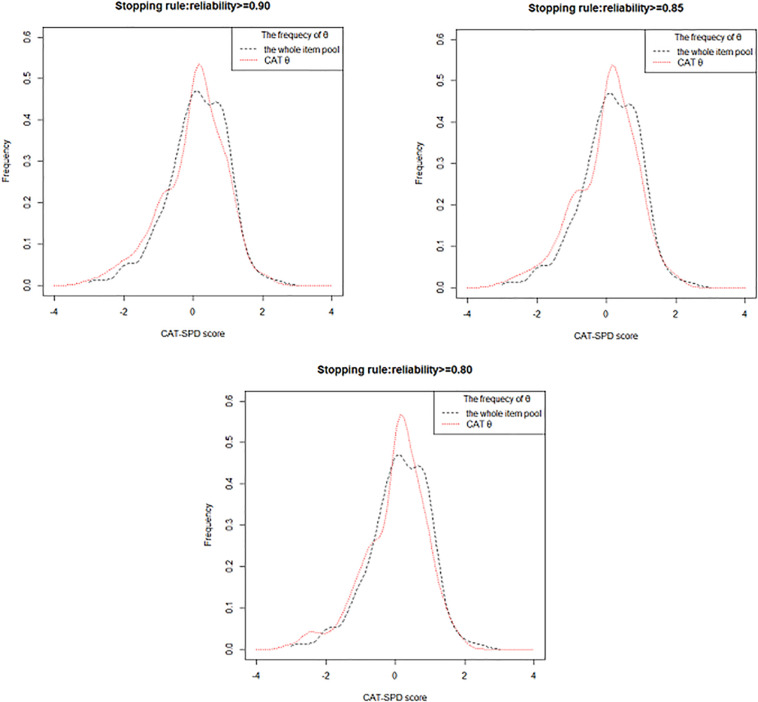
Frequency distribution of the whole item pool and CAT-SPD score under different stopping rules.

Figure 1 depicts the frequency distribution of θ estimation obtained from the entire item pool and the estimated θ *via* the CAT-SPD under different terminating rules. On the other hand, the frequency distribution of the θ estimator obtained by the two versions becomes more identical, as the measurement precision rises. From the picture, two distributions are relatively analogous, which again illustrates that the CAT-SPD is efficient.

**FIGURE 2:**
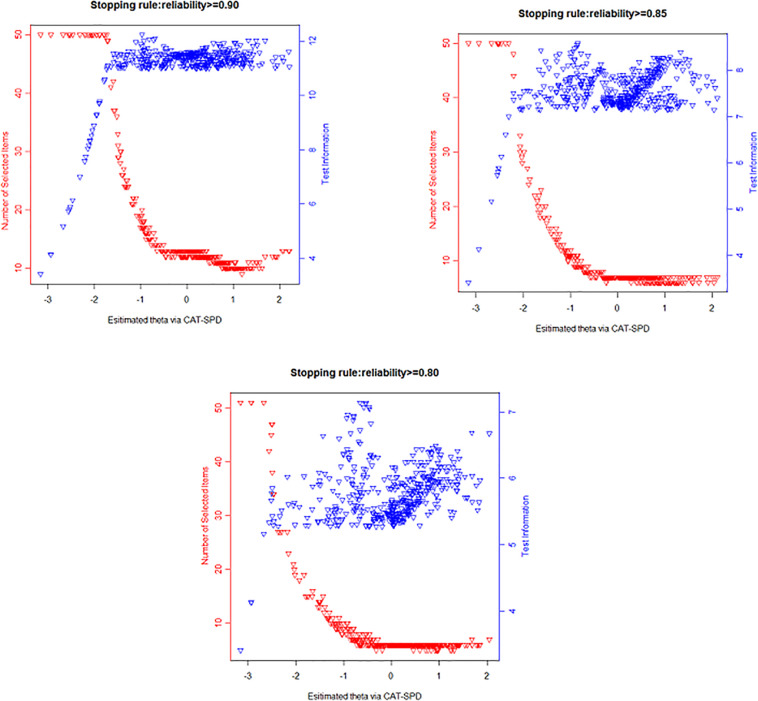
Number of administrated items and test information curve under different stopping rules.

The numbers of item usage along with the test information plots under different terminating rules are presented in Figure 2. Apparently, those plots show the CAT-SPD is informative on the middle or right side of the estimated latent SPD score. Individuals with lower θ estimator administered a large number of items and still have low test information, while fewer items were selected for most respondents with middle or high trait estimations and the testing information is high. For instance, although participants whose theta varied from -3 to -1.5 administered the maximum number of administered items (*N* = 50), their testing information is still low under the terminating rule reliability ≥ 0.90; on the contrary, the testing information exceeded 10 (corresponding to reliability ≥ 0.90) for examinees whose theta covers from 0 to 2.5 with approximately 12 administered items for them.

**FIGURE 3:**
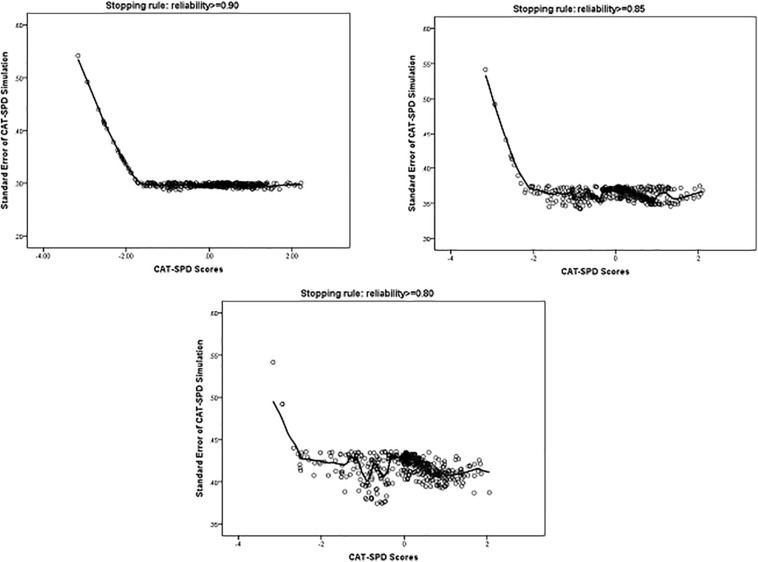
Standard Error (SE) of CAT-SPD score under different stopping rules.

Figure 3 displays the standard error of the estimated trait *via* the CAT-SPD under several stopping rules. As depicted in Figure 3, examinees with middle or high trait estimator have a smaller standard error, which suggests a good measure precision for a wide range of the estimated trait *via* the CAT-SPD.

**FIGURE 4:**
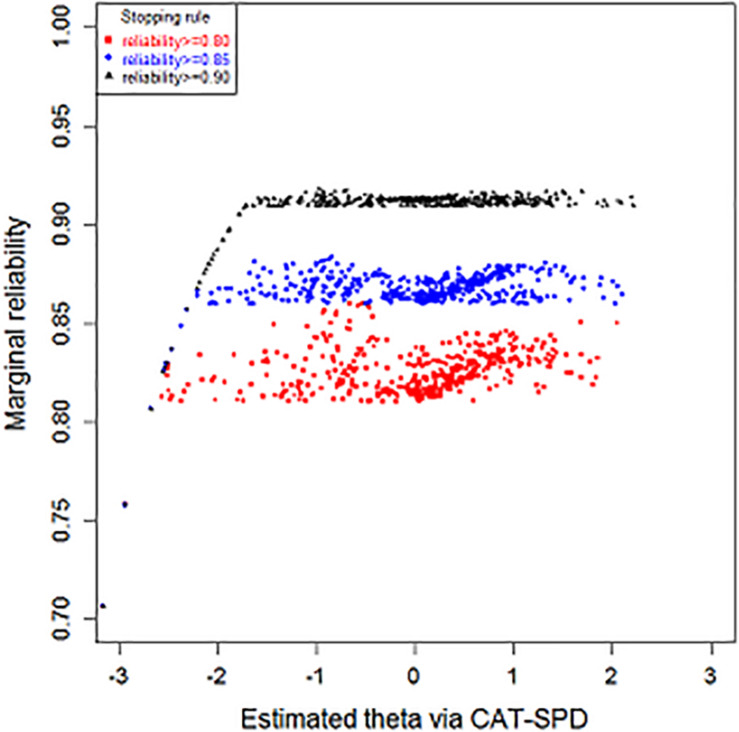
Reliability as a function of the theta under different stopping rules.

Figure 4 and Table 4 display the outcome of marginal reliability of the CAT-SPD. From Table 4, the estimate of marginal reliability in different terminating rules ranges from 0.83 to 0.92, with the mean of 0.87. Figure 4 shows the reliability of subjects with various latent trait levels under different terminating rules. When the CAT was terminated at reliability ≥ 0.90, many trait estimations have high reliabilities above 0.90. Under the stopping rule reliability ≥ 0.85, most individuals’ reliability is higher than 0.85. These outcomes demonstrate that the CAT-SPD developed in this article has good reliability for most participants one more time. What is more, some respondents whose trait scores were over -2 have maximal reliability under the terminating rule reliability ≥ 0.90. When the stopping rules were set at reliability ≥ 0.90 and reliability ≥ 0.85, values of reliability for those with trait scores smaller than -2 are identical. Respondents usually own minimum reliability in the terminating rule reliability ≥ 0.80, no matter where the estimated theta is located.

#### The Content Validity of the CAT-SPD

Based on evaluating items of the item pool by three psychiatrists with more than 5 years of experience, Table 5 displays the distribution of items under each diagnostic criterion in the DSM-5 for the initial and final item pool. As shown in the table, the final item pool covers all nine diagnostic criteria of SPD. The symptom of ideas of reference retained the most items (*N* = 14), while the minimum items (*N* = 6) measured the symptom of magic thinking and odd speech. Each symptom is measured on average by 10 items. In addition, the number of items reserved under individual diagnostic criterion is relatively uniform. These results reveal that the final item pool for the CAT-SPD has acceptable content validity.

**TABLE 5:** The distribution of items in each diagnostic criterion of SPD in DSM-5.

Diagnostic criterion	Initial item pool	Final item pool
Ideas of reference	1, 10, 18, 27, 36, 44, 52, 59, 62, 73, 75, 76, 77, 78, 79, 80, 81, 82, 83, 84, 85, 86	10, 18, 27, 36, 44, 59, 62, 75, 78, 79, 80, 84, 85, 86
Excessive social anxiety	2, 11, 19, 28, 37, 45, 53, 69, 95, 100, 106, 109, 110, 112 113, 118, 123, 124	2, 11, 28, 37, 69, 95, 100, 106, 113, 118, 124
Magic thinking	3, 12, 20, 29, 38, 46, 54, 89, 94, 99, 103, 104, 117, 120, 121, 127	46, 94, 103, 117, 121, 127
Unusual perceptual experience	4, 13, 21, 30, 39, 47, 55, 60, 63, 87, 93, 101, 108, 115	4, 13, 30, 39, 47, 55, 60, 63, 87, 93, 101, 108, 115
Odd behavior	5, 14, 22, 31, 65, 68, 72, 88, 98, 102, 116, 126	5, 14, 22, 31, 65, 68, 72, 88, 98, 102, 116
No close friends	6, 15, 23, 32, 40, 48, 56, 61, 64, 91, 97, 107, 114, 125	15, 23, 32, 40, 48, 56, 64, 91, 97, 107, 114, 125
Odd speech	7, 16, 24, 33, 41, 49, 57, 67, 70, 74	7, 49, 57, 67, 70, 74
Constricted affect	8, 25, 34, 42, 50, 66, 71, 92, 96, 119	8, 34, 42, 50, 66, 71, 92, 119
Suspicious	9, 17, 26, 35, 43, 51, 58, 90, 105, 111, 122, 128	9, 17, 26, 35, 43, 51, 58, 111, 128

#### The Criterion-Related Validity of the CAT-SPD

Pearson’s correlation between the CAT-SPD theta estimates and the schizotypal score of the PDQ-4 (Hyler, 1994) was computed to explore the criterion-related validity of the CAT-SPD. Pearson’s correlation between theta estimations *via* the whole item pool and scores of the schizotypal subscale in the PDQ-4 is 0.62 (*p* ≤ 0.01). Under the terminating rules of reliability ≥ 0.90, reliability ≥ 0.85, and reliability ≥ 0.80, the Pearson’s correlations are 0.58 (*p* ≤ 0.01), 0.55 (*p* ≤ 0.01), and 0.54, (*p* ≤ 0.01), respectively, which demonstrated that the criterion-related validity of the CAT-SPD is basically acceptable.

#### The Predictive Utility (Sensitivity and Specificity) of the CAT-SPD

Table 6 displays the ROC analysis results. These statistics reveal the detection performance of the CAT-SPD. For the AUC, the value is the highest with 0.87 (sensitivity = 0.913, specificity = 0.695) when no stopping rule was applied. When the terminating rule was set at reliability ≥ 0.90, reliability ≥ 0.85, and reliability ≥ 0.80, respectively, the values of AUC are 0.802 (sensitivity = 0.826, specificity = 0.704), 0.793 (sensitivity = 0.783, specificity = 0.757), and 0.792 (sensitivity = 0.761, specificity = 0.75), respectively. Besides, the Youden index (Youden, 1950) as a common metric was used to assess sensitivity and specificity. The entire item pool has the largest value of Youden index with 0.608, followed by the stopping rule reliability ≥ 0.90. The two lowest Youden indices are the terminating rule reliability ≥ 0.85 and reliability ≥ 0.80, with 0.54 and 0.511. These results also suggest that the significant decrease of time administered and the number of items using the CAT format bring about only a less drop in prediction precision.

**TABLE 6:** The predictive utility (sensitivity and specificity) of the CAT-SPD under different terminating rules.

Stopping rule	PDQ-4			
	AUC (95% CI)	Se	Sp	YI
None	0.872 (0.850–0.918)	0.913	0.695	0.608
Reliability ≥ 0.80	0.792 (0.726–0.860)	0.761	0.750	0.511
Reliability ≥ 0.85	0.793 (0.714–0.862)	0.783	0.757	0.540
Reliability ≥ 0.90	0.802 (0.741–0.875)	0.826	0.704	0.530

*95% CI, 95% confidence interval; None, the whole item pool was administered; AUC, area under the curve; Se, sensitivity; Sp, specificity; YI, Youden index.*

## Discussion

The goal of this study was to develop an accurate and effective CAT version for SPD (CAT-SPD). Toward this end, the research first constructed an item pool with high quality for CAT-SPD based on DSM-5 and a series of IRT analyses. Then, the performance of the CAT-SPD was evaluated in a simulated environment based on participants’ real responses. The results indicated that the CAT-SPD had an acceptable performance which was embodied in the following aspects. (1) The item pool with 90 items for the CAT-SPD had good characteristics, embodied by evidence for sufficient unidimensionality, local independence, good item model fit, absence of DIF, and high average discrimination (*a* = 1.22); (2) consistency ranging from 0.85 to 0.95 of the trait scores (the CAT simulation vs. the full item pool) was high for all applied stopping rules; and (3) detecting performance with regard to its ability to screen individuals at risk for SPD (AUC = 0.872 for the full item pool) was basically the idea. The results in this study showed that the termination criterion reliability ≥ 0.90 (corresponding to *SE* ≤ 0.316) would be an optimal choice for the CAT-SPD in that the number of item usage was low and trait estimations *via* the CAT showed high congruence with trait scores through the whole item pool.

Compared with the lately developed CAT for schizotypy (Fonseca-Pedrero et al., 2013; Moore et al., 2018), the new CAT-SPD has potentially remarkable attributes as follows: (1) A larger item pool with high quality was constructed based on the diagnostic criteria of SPD in the DSM-5 and three well-validated psychological sales, which may provide more choices for respondents with different ability levels when selecting an item. (2) A comprehensive DIF analysis for gender and region was carried out in the process of developing the item pool for SPD. However, no DIF analysis was performed in the study of Moore et al. (2018). Hence, nothing could be said regarding how far the measurement tool functions independently of, e.g., gender or region. (3) This study compared four commonly used IRT models with polytomous scoring in the CAT, then an optimal model was chosen to fit to the CAT-SPD based on the test-level model-fit test. (4) Because applying the identical sample to calibrate item and simulate the CAT may supply a flattering outcome, cross-validation was performed to obtain more objective and scientific results in this study.

Although the current article demonstrated a great potential that CAT-SPD could increase the efficiency of SPD assessment, when applying CAT-SPD in practice, practitioners and researchers should clarify whether the CAT format here is suitable for their assessment objective. The test information curve (Figure 2) displayed that the information of CAT-SPD peaks on the right side of the trait continuum. Hence, for individuals with a similar level of SPD, a small discrepancy could be more easily screened for participants with high trait estimations compared with participants with low trait estimations. That is typical in the area of clinical assessment and IRT (Waller and Reise, 1989). It could be ascribed to the fact that the psychopathology structure might be unipolar (Waller and Reise, 1989). For a certain measurement scenario, measurement accuracy should distribute evenly rather than distribute in peaks in the scale. If someone intends to apply CAT-SPD to an analogous case, new items with extremely low location parameter (e.g., more easily endorsed items) should be expanded to the item pool. As for the other scenarios, SPD assessment might be specialized for deciding whether a respondent could be diagnosed with SPD. In such a situation, the CAT-SPD developed in this research is perhaps not an optimal choice. The SPD is a complicated construction which is closely associated with genetic, neurodevelopmental, neurocognitive, social, emotional, and psychophysiological levels to psychotic disorders (Raine, 2006). It is suggested that the diagnosis of SPD could be conducted in multistage progress. In the first stage, the CAT-SPD could serve to detect risky respondents in a rapid and accurate manner. In the following phase, all psychological and medical assessments (e.g., genetic liability, disease history) are required to aid in the diagnosis. When users are satisfied with the test information displayed in the current research, they could make use of CAT as a tool for effective SPD measurement.

This study has some limitations. However, these limitations can provide direction for future research. The deficiencies of the current article are as follows: First, CAT-SPD has an intermediate criterion-related validity for all applied termination criteria and the whole item pool. It implied that the psychosis spectrum categorization applied in this study as a validity criterion may be suboptimal (Moore et al., 2018). Thus, it is recommended that the follow-up studies should use multiple validity scales to analyze the validity of the CAT. Second, item selection strategy is an important component of CAT. Van der Linden (2000) and Cheng (2009) mentioned that CAT item selection not only considers statistical optimization problems (e.g., the accuracy of assessment), but also meets some non-statistical constraints (e.g., content balance). In the simulation of the CAT-SPD, we used the MFI item selection strategy to improve the measurement accuracy, which might result in an unbalanced number of nine diagnostic criteria being asked for most of the participants. Future research should consider using an item selection strategy, which can improve the accuracy of the tests and consider non-statistical constraints. Third, another shortcoming of the current article is that the performance of the CAT-SPD was evaluated by a simulated CAT rather than a real CAT administration. To assess the performance comprehensively, a field test can be conducted on the subjects by developing a real CAT administration for the CAT-SPD.

## Data Availability Statement

The original contributions presented in the study are included in the article/Supplementary Material. Further inquiries can be directed to 1246213148@qq.com.

## Ethics Statement

The studies involving human participants were reviewed and approved by the Research Center of Mental Health of Jiangxi Normal University. The participants provided their written informed consent to participate in this study.

## Author Contributions

YL was responsible for data processing and manuscript writing. DT and YC contributed to the manuscript’s revision. MS was responsible for the data collection. All authors contributed to the article and approved the submitted version.

## Conflict of Interest

The authors declare that the research was conducted in the absence of any commercial or financial relationships that could be construed as a potential conflict of interest.
