# Supplementary material for: Computerized Adaptive Testing for Schizotypal Personality Disorder: Detecting Individuals at Risk
Source: Front Psychol. 2021 Jan 25;11:574760. doi: 10.3389/fpsyg.2020.574760 (PMC7868333; doi:10.3389/fpsyg.2020.574760)
Supplement: Supplementary file 1 [file Data_Sheet_1.docx]

Supplementary Material

# Supplementary Figures and Tables

**Table** | IRT statistics of items in the final item pool of CAT-SPD

| **Item** | **Abbreviated item content** | **Scale** | **Item parameter** | | | | | **Item-fit estimates** | | | | ***R^2^*change** | | **Diagnostic criterion** |
| --- | --- | --- | --- | --- | --- | --- | --- | --- | --- | --- | --- | --- | --- | --- |
|  |  |  | **Slope** | **b1** | **b2** | **b3** | **b4** | | ***S-X^2^*** | ***df*** | ***p*** | | ***DIF*** |  |
| 2 | Avoid crowds due to anxiety | SPQ | 0.58 | -0.07 | - | - | - | | 72.17 | 80 | 0.722 | | 0.0052 | Social Anxiety |
| 4 | Mistaken objects for people | SPQ | 0.61 | 1.29 | - | - | - | | 56.87 | 73 | 0.918 | | 0.0001 | Unusual Per Experience |
| 5 | See me as eccentric | SPQ | 1.69 | 1.40 | - | - | - | | 32.84 | 43 | 0.869 | | 0.0025 | Odd Behavior |
| 7 | Hard understand my word | SPQ | 1.45 | 0.63 | - | - | - | | 60.27 | 64 | 0.609 | | 0.0024 | Odd Speech |
| 8 | Someone feels I am cold | SPQ | 0.82 | 0.61 | - | - | - | | 73.61 | 74 | 0.491 | | 0.0021 | Constricted Affect |
| 9 | Sure I being talked behind me | SPQ | 1.07 | 1.42 | - | - | - | | 48.54 | 59 | 0.833 | | 0.0008 | Suspicious |
| 10 | Fell like people notice me | SPQ | 0.87 | 1.33 | - | - | - | | 51.59 | 67 | 0.918 | | 0.0015 | Ideas Reference |
| 11 | Get nervous interacting with others | SPQ | 0.70 | 0.70 | - | - | - | | 72.97 | 74 | 0.512 | | 0.0006 | Social Anxiety |
| 13 | Sense force around you | SPQ | 0.89 | 1.40 | - | - | - | | 71.50 | 65 | 0.271 | | 0.0036 | Unusual Per Experience |
| 14 | Comment my unusual mannerisms | SPQ | 1.16 | 0.72 | - | - | - | | 73.14 | 67 | 0.283 | | 0.0124 | Odd Behavior |
| 15 | Keep myself to myself | SPQ | 1.53 | 1.47 | - | - | - | | 37.63 | 43 | 0.703 | | 0.007 | No Friends |
| 17 | Feel others making trouble for me | SPQ | 1.30 | 1.53 | - | - | - | | 54.53 | 49 | 0.272 | | 0.0114 | Suspicious |
| 18 | Will someone give you some hints | SPQ | 0.57 | -0.16 | - | - | - | | 70.76 | 81 | 0.785 | | 0.0048 | Ideas Reference |
| 22 | Others think me strange | SPQ | 1.70 | 0.84 | - | - | - | | 45.85 | 57 | 0.855 | | 0.0049 | Odd Behavior |
| 23 | Hardly speak I being with others | SPQ | 0.97 | 1.79 | - | - | - | | 56.37 | 53 | 0.35 | | 0.012 | No Friends |
| 26 | Worry friends being unfaithful | SPQ | 0.95 | 0.34 | - | - | - | | 71.28 | 72 | 0.502 | | 0.0091 | Suspicious |
| 27 | Treat common things as special sign | SPQ | 0.92 | 0.57 | - | - | - | | 65.97 | 71 | 0.647 | | 0.0065 | Ideas Reference |
| 28 | Feel anxious meeting someone | SPQ | 0.89 | 0.27 | - | - | - | | 78.31 | 74 | 0.344 | | 0.003 | Social Anxiety |
| 30 | Hear a voice speaking my thoughts | SPQ | 1.52 | 1.43 | - | - | - | | 37.21 | 45 | 0.789 | | 0.0003 | Unusual Per Experience |
| 31 | Think me as bizarre person | SPQ | 2.21 | 1.19 | - | - | - | | 34.08 | 40 | 0.733 | | 0.0017 | Odd Behavior |
| 32 | Difficult get close to others | SPQ | 1.00 | 0.69 | - | - | - | | 76.33 | 70 | 0.282 | | 0.0029 | No Friends |
| 34 | Bad nonverbal communication | SPQ | 0.89 | 1.39 | - | - | - | | 64.09 | 65 | 0.509 | | 0.0157 | Constricted Affect |
| 35 | Being vigilant I being with friends | SPQ | 1.20 | 1.24 | - | - | - | | 56.70 | 60 | 0.597 | | 0.0094 | Suspicious |
| 36 | See special meaning in ads | SPQ | 0.80 | 1.03 | - | - | - | | 74.08 | 72 | 0.41 | | 0.0002 | Ideas Reference |
| 37 | Feel nervous when in unfamiliar people | SPQ | 0.66 | -0.29 | - | - | - | | 85.33 | 77 | 0.241 | | 0.0038 | Social Anxiety |
| 39 | See things invisible to other | SPQ | 1.59 | 1.80 | - | - | - | | 26.81 | 32 | 0.727 | | 0.0018 | Unusual Per Experience |
| 40 | Haven’t close friend | SPQ | 0.98 | 1.14 | - | - | - | | 69.22 | 66 | 0.369 | | 0.0071 | No Friends |
| 42 | Poor for social etiquette | SPQ | 1.02 | 0.64 | - | - | - | | 71.07 | 70 | 0.442 | | 0.0035 | Constricted Affect |
| 43 | Feel hidden threats | SPQ | 1.50 | 0.95 | - | - | - | | 69.05 | 58 | 0.152 | | 0.0002 | Suspicious |
| 44 | Feel others notice me when shopping | SPQ | 1.23 | 1.17 | - | - | - | | 53.92 | 60 | 0.696 | | 0.0011 | Ideas Reference |
| 46 | Have experiences with astrology | SPQ | 0.67 | 1.93 | - | - | - | | 55.42 | 65 | 0.796 | | 0.0152 | Magic Thinking |
| 47 | See daily things large or small | SPQ | 1.11 | 0.69 | - | - | - | | 73.88 | 69 | 0.322 | | 0.0004 | Unusual Per Experience |
| 48 | Not worth for writing to friend | SPQ | 1.00 | 1.65 | - | - | - | | 64.38 | 56 | 0.207 | | 0.0147 | No Friends |
| 49 | Use words in unusual ways | SPQ | 0.88 | 0.12 | - | - | - | | 72.71 | 74 | 0.521 | | 0.002 | Odd Speech |
| 50 | Avoid eye contact | SPQ | 1.12 | 0.60 | - | - | - | | 58.45 | 69 | 0.813 | | 0.0009 | Constricted Affect |
| 51 | Know much about you is bad | SPQ | 0.84 | 0.02 | - | - | - | | 74.92 | 75 | 0.481 | | 0.0002 | Suspicious |
| 55 | Feel strong sense of smell | SPQ | 0.73 | 0.86 | - | - | - | | 96.06 | 73 | 0.037 | | 0.0095 | Unusual Per Experience |
| 56 | Keep silent in social situations | SPQ | 0.90 | 0.15 | - | - | - | | 59.82 | 74 | 0.884 | | 0.0062 | No Friends |
| 57 | Wander off the topic talking with others | SPQ | 0.95 | 1.21 | - | - | - | | 57.66 | 66 | 0.758 | | 0.0087 | Odd Speech |
| 58 | Feel others bear me a grudge | SPQ | 1.48 | 1.62 | - | - | - | | 48.37 | 41 | 0.2 | | 0.0117 | Suspicious |
| 59 | Fell someone watching me | SPQ | 0.74 | -0.12 | - | - | - | | 84.69 | 76 | 0.232 | | 0.0003 | Ideas Reference |
| 60 | Distracted by distant sounds | SPQ | 1.02 | 0.44 | - | - | - | | 83.50 | 69 | 0.113 | | 0.0094 | Unusual Per Experience |
| 62 | Feel someone talking about me | SPQ | 0.96 | 0.75 | - | - | - | | 44.20 | 71 | 0.995 | | 0.0037 | Ideas Reference |
| 63 | Hear my strong thoughts | SPQ | 1.37 | 1.23 | - | - | - | | 66.43 | 56 | 0.161 | | 0.0029 | Unusual Per Experience |
| 64 | Cannot get close to people | SPQ | 1.90 | 1.33 | - | - | - | | 50.71 | 41 | 0.142 | | 0.0039 | No Friends |
| 65 | I am an unusual person | SPQ | 1.99 | 1.24 | - | - | - | | 28.47 | 39 | 0.893 | | 0.0103 | Odd Behavior |
| 66 | I have an inactive way of speech | SPQ | 0.91 | 1.14 | - | - | - | | 93.60 | 67 | 0.018 | | 0.0018 | Constricted Affect |
| 67 | Hard communicate clearly | SPQ | 1.47 | 0.97 | - | - | - | | 76.62 | 58 | 0.051 | | 0.0026 | Odd Speech |
| 68 | Have some weird habits | SPQ | 1.32 | 0.92 | - | - | - | | 83.37 | 62 | 0.037 | | 0.006 | Odd Behavior |
| 69 | Feel uneasy contracting with others | SPQ | 1.01 | 0.24 | - | - | - | | 79.51 | 70 | 0.204 | | 0.0103 | Social Anxiety |
| 70 | My speech is confused | SPQ | 1.10 | 0.87 | - | - | - | | 84.76 | 67 | 0.07 | | 0.0027 | Odd Speech |
| 71 | Repress emotion | SPQ | 0.98 | 0.11 | - | - | - | | 55.30 | 70 | 0.9 | | 0.0006 | Constricted Affect |
| 72 | Stare me due to odd appearance | SPQ | 1.16 | 1.92 | - | - | - | | 44.27 | 44 | 0.46 | | 0.0164 | Odd Behavior |
| 74 | Difficultly understand my speech | REF | 1.58 | 1.61 | - | - | - | | 46.17 | 37 | 0.143 | | 0.0092 | Odd Speech |
| 75 | Wonder laughing at me | REF | 1.38 | 1.42 | - | - | - | | 60.31 | 49 | 0.129 | | 0.002 | Ideas Reference |
| 78 | Have similar experience with film | REF | 0.69 | 1.51 | - | - | - | | 83.38 | 70 | 0.131 | | 0.0094 | Ideas Reference |
| 79 | Feel watching me when taking the bus | REF | 0.92 | 2.07 | - | - | - | | 46.93 | 51 | 0.636 | | 0.0068 | Ideas Reference |
| 81 | Animas noticing me when walking | REF | 1.05 | 2.17 | - | - | - | | 39.93 | 42 | 0.562 | | 0.0054 | Ideas Reference |
| 84 | Others notice my hided personality | REF | 1.32 | 1.71 | - | - | - | | 40.77 | 43 | 0.568 | | 0.0063 | Ideas Reference |
| 85 | Wonder blame me | REF | 1.06 | 1.57 | - | - | - | | 50.01 | 56 | 0.7 | | 0.001 | Ideas Reference |
| 86 | Condemn behavior | REF | 1.67 | 1.38 | - | - | - | | 56.96 | 44 | 0.091 | | 0.0014 | Ideas Reference |
| 87 | Feel my body unusual | FFSI | 1.17 | -1.04 | 0.29 | 1.74 | 3.76 | | 130.11 | 126 | 0.383 | | 0.0014 | Unusual Per Experience |
| 88 | Think my action odd | FFSI | 1.42 | -0.04 | 1.39 | 2.67 | 3.20 | | 105.22 | 89 | 0.115 | | 0.0106 | Odd Behavior |
| 91 | Don’t form strong bonds | FFSI | 1.38 | -0.65 | 0.82 | 1.59 | 2.99 | | 112.38 | 116 | 0.578 | | 0.0001 | No Friends |
| 92 | Have little to do with other | FFSI | 1.21 | -0.43 | 0.89 | 2.01 | 3.63 | | 106.96 | 120 | 0.797 | | 0.0018 | Constricted Affect |
| 93 | feel body becoming misshapen | FFSI | 1.66 | -0.20 | 0.80 | 1.66 | 2.79 | | 104.48 | 109 | 0.605 | | 0.0015 | Unusual Per Experience |
| 94 | Have odd thinking | FFSI | 1.48 | -1.08 | 0.03 | 1.15 | 2.54 | | 162.96 | 135 | 0.051 | | 0.001 | Magic Thinking |
| 95 | Social occasion making me anxious | FFSI | 1.42 | -1.37 | -0.33 | 0.74 | 2.26 | | 141.21 | 136 | 0.362 | | 0.0063 | Social Anxiety |
| 97 | I like to be alone | FFSI | 1.10 | -2.06 | -0.79 | 0.74 | 2.72 | | 159.71 | 134 | 0.064 | | 0.0131 | No Friends |
| 98 | Describe my behaviors as unusual | FFSI | 2.19 | -0.43 | 0.79 | 1.63 | 2.87 | | 78.69 | 90 | 0.797 | | 0.0025 | Odd Behavior |
| 100 | Feel uneasy with familiar people | FFSI | 1.90 | -0.59 | 0.92 | 1.56 | 2.53 | | 104.70 | 100 | 0.354 | | 0.0093 | Social Anxiety |
| 101 | Sense sometimes is odd | FFSI | 1.88 | -0.51 | 0.83 | 1.61 | 2.87 | | 119.00 | 101 | 0.107 | | 0.0012 | Unusual Per Experience |
| 102 | Say and do odd things | FFSI | 2.37 | -0.42 | 0.81 | 1.52 | 3.05 | | 78.71 | 86 | 0.699 | | 0.0041 | Odd Behavior |
| 103 | Curious if my idea is crazy | FFSI | 1.13 | -1.70 | -0.47 | 0.77 | 3.32 | | 143.15 | 140 | 0.41 | | 0.0083 | Magic Thinking |
| 106 | Have more social anxiety | FFSI | 1.20 | -1.78 | -0.25 | 1.27 | 2.78 | | 117.19 | 126 | 0.701 | | 0.0059 | Social Anxiety |
| 107 | Like to make lots of friends | FFSI | 0.59 | -3.26 | 0.18 | 2.59 | 5.19 | | 112.11 | 132 | 0.894 | | 0.0067 | No Friends |
| 108 | Have strange experiences | FFSI | 1.08 | -1.28 | 0.45 | 1.82 | 3.44 | | 154.93 | 128 | 0.053 | | 0.0003 | Unusual Per Experience |
| 111 | Feel others having it in for me | FFSI | 1.18 | -1.13 | 0.56 | 1.90 | 3.60 | | 128.77 | 123 | 0.343 | | 0.0006 | Suspicious |
| 113 | Worry embarrass myself | FFSI | 0.82 | -3.16 | -1.25 | 0.41 | 2.76 | | 124.47 | 140 | 0.822 | | 0.0079 | Social Anxiety |
| 114 | Don’t touch friend | FFSI | 1.02 | -1.35 | 0.51 | 1.53 | 3.95 | | 137.50 | 142 | 0.591 | | 0.0072 | No Friends |
| 115 | have weird felling | FFSI | 1.52 | -1.16 | -0.17 | 0.92 | 2.78 | | 135.98 | 132 | 0.388 | | 0.0001 | Unusual Per Experience |
| 116 | Know I am strange in the eyes of others | FFSI | 2.20 | -0.51 | 0.55 | 1.37 | 2.35 | | 75.43 | 97 | 0.949 | | 0.0029 | Odd Behavior |
| 117 | Like studying odd belief | FFSI | 1.48 | -0.50 | 0.78 | 1.74 | 2.99 | | 135.56 | 114 | 0.082 | | 0.0018 | Magic Thinking |
| 118 | Feel I leaving a bad impression | FFSI | 1.43 | -1.32 | -0.04 | 1.18 | 3.12 | | 121.80 | 123 | 0.514 | | 0.0015 | Social Anxiety |
| 119 | I am an indifferent person | FFSI | 1.50 | -0.84 | 0.43 | 1.47 | 2.86 | | 139.06 | 117 | 0.08 | | 0.002 | Constricted Affect |
| 121 | Have odd thoughts | FFSI | 2.04 | -0.62 | 0.46 | 1.35 | 2.43 | | 99.85 | 101 | 0.514 | | 0.0009 | Magic Thinking |
| 124 | Be awkward around people | FFSI | 1.60 | -1.25 | 0.24 | 1.37 | 2.73 | | 116.63 | 108 | 0.268 | | 0.0065 | Social Anxiety |
| 125 | Little friend | FFSI | 1.18 | -1.79 | -0.16 | 0.90 | 2.53 | | 161.84 | 141 | 0.11 | | 0.0028 | No Friends |
| 127 | Believe unusual things | FFSI | 1.10 | -1.18 | 0.49 | 1.46 | 3.23 | | 140.86 | 145 | 0.582 | | 0.0004 | Magic Thinking |
| 128 | Safe to keep to yourself | FFSI | 0.84 | -2.89 | -1.40 | 0.15 | 2.36 | | 143.62 | 153 | 0.695 | | 0.001 | Suspicious |

*Note: SPQ is the Schizotypal Personality Questionnaire; REF is the Referential Thinking Scale. FFSI refers to the Five-Factor Schizotypal Inventory.*
